# Potential Application of Innovative *Aspergillus terreus*/Sodium Alginate Composite Beads as Eco-Friendly and Sustainable Adsorbents for Alizarin Red S Dye: Isotherms and Kinetics Models

**DOI:** 10.3390/microorganisms11051135

**Published:** 2023-04-27

**Authors:** Aya I. Tagyan, Manal M. Yasser, Ahmed M. Mousa, Dalal Hussien M. Alkhalifah, Wael N. Hozzein, Marym A. Marzouk

**Affiliations:** 1 Department of Botany and Microbiology, Faculty of Science, Beni-Suef University, Beni-Suef 62511, Egypt; i_aya50@yahoo.com (A.I.T.); manal_yaser2006@yahoo.com (M.M.Y.); ashmmousa@yahoo.com (A.M.M.); marym.marzouk@yahoo.com (M.A.M.); 2 Department of Biology, College of Science, Princess Nourah bint Abdulrahman University, P.O. Box 84428, Riyadh 11671, Saudi Arabia

**Keywords:** *Aspergillus terreus*, sodium alginate, adsorption, Alizarin Red S, isotherm, kinetic, bioremediation, fungi

## Abstract

Fungi were used as one of the most common bioremediation methods. From this perspective, our study highlights the optimization of Alizarin Red S (ARS) dye adsorption performance for the sodium alginate (SA) by using the fungus *Aspergillus terreus* (*A. terreus*) to form a composite bead and the possibility of its reusability. This was accomplished by mixing SA with different ratios of biomass powder of *A. terreus,* including 0%, 10%, 20%, 30%, and 40%, to form composite beads of *A. terreus*/SA-0%, *A. terreus*/SA-10%, *A. terreus*/SA-20%, *A. terreus*/SA-30%, and *A. terreus*/SA-40%, respectively. The ARS adsorption characteristics of these composite mixtures were analyzed at various mass ratios, temperatures, pH values, and initial concentrations. Moreover, sophisticated techniques, such as scanning electron microscopy (SEM) and Fourier-transform infrared spectroscopy (FTIR), were employed to detect the morphological and chemical properties of this composite, respectively. The experimental results revealed that *A. terreus*/SA-20% composite beads have the highest adsorption capacity of 188 mg/g. Its optimum adsorption conditions were achieved at 45 °C and pH 3. Moreover, the ARS adsorption was well explained by the Langmuir isotherm (q_m_ = 192.30 mg/g) and pseudo-second-order and intra-particle diffusion kinetics. The SEM and FTIR findings corroborated the superior uptake of A. terreus/SA-20% composite beads. Lastly, the *A. terreus*/SA-20% composite beads can be employed as an eco-friendly and sustainable alternative to other common adsorbents for ARS.

## 1. Introduction

Colorful organic dyes are extensively utilized in numerous industries, such as textiles, food coloring, cosmetics, and printing [1,2]. Without continuous treatment, plenty of dyes are poured into the wastewater, causing severe environmental damage, such as an increase in water turbidity, biological oxygen demand (BOD), chemical oxygen demand (COD), and sunlight block [3]. Anthraquinone dyes are the second most important industrially necessary colors due to their rapidity and remarkable photosensitive stability [4]. These dyes contain two chromophore and auxochrome groups (i.e., −COOH, −OH, −NO_2_, C=O, and C=C), which can be directly adsorbed onto hydrophobic and fibrous materials to increase the color and electrolytic dissolution, respectively [5]. These groups are responsible for the wavelength, absorption, and binding strength of colors.

Nevertheless, these dyes resist degradation due to their fused aromatic structures [6]. Alizarin Red S (ARS; polar) is a common anthraquinone dye with a considerable market value in the textile industry [7]. ARS is a dye that has been used in various industries for centuries, including the textile industry. It is a synthetic organic compound that has a red color and is commonly used for a variety of applications, including textile dyeing, printing, and staining in biology and histology. Currently, ARS is primarily used in the research field, particularly in the study of bone tissue and osteoporosis. It is used to stain calcium deposits in bone tissue and analyze bone density and structure. In terms of market size, alizarin is a relatively small segment of the overall dye and pigment market. Overall, while alizarin still has some niche applications in research, its use in industry and consumer products has declined due to environmental and health concerns [8]. More specifically, ARS is a carcinogenic, mutagenic, anionic, long-lasting, and persistent dye that affects biological creatures. Therefore, it is crucial to discover an economical and environmentally suitable treatment procedure before releasing it into the environment. For dye remediation, numerous physicochemical methods, including activated carbon, electro-oxidation, chemical oxidation, photolysis, flocculation, and bioremediation, can be used [9,10,11,12,13,14,15,16]. Bioremediation can be applied using microorganisms such as algae, bacteria, and fungi [17]. Studies showed that bioremediation by fungi is a practical, appropriate, and eco-friendly procedure [18,19]. Ascomycetes are typical fungi that are essential in bioremediation as they are fast-growing and low in cost [20,21]. Overall, the bioremediation of pollutants by fungi occurs through biosorption, adsorption, and enzymatic approaches [22]. More specifically, the fungal cell walls contain polysaccharides (chitin, chitosan, and glucan), proteins, lipids, and melanin. Fungi have different functional groups, for example, RNH_2_, COOH, and PO₄³⁻, that can bind dye molecules [23,24]. Previous research has studied the binding mechanisms of contaminants with diverse reactive groups and molecular structures [25]. Acid dye biosorption involves electrostatic interactions, which can significantly cause dye discoloration. As a result, the functional groups that have a positive charge on the fungal surface can attract anthraquinone dyes that are negatively charged [26].

Furthermore, the groups of amines in the biomass of fungi are protonated after the pH of dye values is adjusted with HCl [26]. Therefore, these protonated amino groups facilitate colored biosorption [27]. On the other hand, sodium alginate (SA) is a negatively charged polymer and natural polysaccharide [28], which is extracted from Phaeophyceae [29,30]. SA has functional groups such as OH and COOH. When cross-linking agents of divalent metal cations (e.g., CaCl_2_) are added to the SA solution, hydrogels, membranes, and beads can be formed [31]. Thus, the composites of SA beads are easily manufactured, economical, and eco-friendly materials. In numerous studies, ARS has been successfully adsorbed by various adsorbents such as carbon nanotubes, activated carbon, activated clay modified by iron oxide, and graphene oxide/attapulgite composite [32,33,34]. To our knowledge, *A. terreus*/SA composite beads have not been considered for the remediation of ARS before. Hence, the current study focuses on increasing the ARS adsorption capacity of SA by mixing it with *A. terreus* in different ratios to prepare *A. terreus*/SA composite beads. The ideal sample was nominated based on the adsorption tests conducted for different mass ratios of *A. terreus* (0%, 10%, 20%, 30%, and 40%) to SA. After that, the adsorption efficiency of this optimum ratio of *A. terreus*/SA composite beads was investigated at different parameters, including pH, temperature, contact time, and initial ARS concentration. Also, the reusability of this sample was discussed. Moreover, the desorption parameters of kinetics and isotherms, as well as the mechanism, were also investigated for the nominated sample of *A. terreus*/SA composite beads. The surface morphology and functional groups of these composite beads were assessed using scanning electron microscopy (SEM) and Fourier-transform infrared spectroscopy (FTIR) to validate the desorption findings.

## 2. Materials and Methods

### 2.1. Reagents

Sodium alginate (SA) was obtained from FoodChem International Corporation (El Monte, CA, USA). The calcium chloride (CaCl_2_), hydrochloric acid (HCl), and sodium hydroxide (NaOH) were all purchased from PioChem (Giza, Egypt). Alizarin Red S (ARS) was obtained from Loba Chemie (Mumbai, India). Distilled water was used in the work.

### 2.2. Isolation and Identification of the Fungal Strain

The fungus was isolated from textile industrial effluents following the methodology described previously [35]. The pure fungus was identified based on three techniques: colony morphology and microscopic and molecular characteristics. The fungus was isolated on PDA media plates containing 250 mg/L chloramphenicol, and then these plates were incubated at 28 °C to study the colony morphology. The emerging fungus from the wastewater sample was monitored every 2 days for 5 days. The mycelia isolated from this sample were separated regularly, and the hyphal tips were transferred to antibiotic-free PDA plates. Then, the single-spore cultures were prepared to confirm the purity of the strain. The fungal colonies were examined after incubation at 28 °C in different media for 5 days, involving PDA, malt extract agar (MEA), and Sabouraud dextrose agar (SDA). Then, the isolates were examined under an optical microscope to verify each conidial head, conidiophore, vesicle, and conidia.

As for the molecular identification of the fungus, polymerase chain reaction (PCR) was used to amplify the 18S rRNA gene from the fungal DNA. The amplified product was subjected to Sanger sequencing, and the sequence was obtained. After that, it was entered into the GenBank NCBI database, and their homology was ascertained using a BLASTn search at https://www.ncbi.nlm.nih.gov (accessed on 20 February 2023).

### 2.3. Material Preparation

#### 2.3.1. Preparation of Fungal Powder

After the fungus was incubated for 5 days at 28 °C, it was inoculated on potato dextrose broth media. It was killed by adding NaOH (0.5 N) in a conical flask with a fungal mat and keeping it in a water bath for 15 min. After that, the mat was filtrated and washed with distilled water about 5–6 times until the pH reached 7. The mat was then transferred to a sterile plate and dried in the oven for 48 h at 80 °C. Then, the dried mat was examined by optical microscopy and colony-forming unit (CFU) to verify the death of conidia. Following that, the dried mat was ground into smaller fractions using porcelain mortar, as it is known that the smaller the particle size, the larger the surface area, and then sieved to reach a range of 100–150 µm. Later, it was stored in a sterile container for further study.

#### 2.3.2. *A. terreus*/SA Composite Beads Preparation

**Figure 1:**
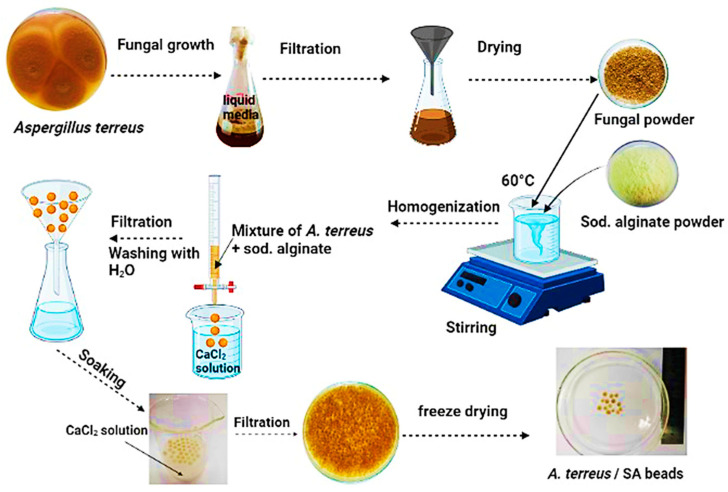
Synthesis method of *A. terreus*/SA composite beads used for ARS uptake.

A hot plate magnetic stirrer was employed to dissolve 1.6 g of SA in 40 mL distilled water for 2 h to prepare a 4% solution of SA. Four ratios of *A. terreus*, including 10%, 20%, 30%, and 40% by composite weight, were designed by taking different weights of *A. terreus*, including 0.16 g, 0.32 g, 0.48 g, and 0.64 g, respectively, for incorporating into the solution of SA with stirring for 6 h to attain a mixture of gel with uniform viscosity. Such ratios, i.e., 10, 20, 30, and 40%, were referred to as *A. terreus*/SA-10%, *A. terreus*/SA-20%, *A. terreus*/SA-30%, and A. *terreus*/SA-40%, respectively. The control sample with zero weight of *A. terreus* (0%) is referred to as SA. For forming gel beads, this gel mix was injected into a 3% solution of CaCl_2_ at 4 °C. Then, the formed beads were soaked in a hardening agent solution of 2.0% Na_2_B_4_O_7_. Subsequently, these beads were maintained in a 3% solution of CaCl_2_ (cross-linking agent) for 24 h. Then, the composite beads were collected and washed with distilled water many times until the impurities on their surface were eliminated. Those beads were left at 25 °C until no water was secreted before being sealed and kept away from air. Finally, the beads were freeze-dried to maintain a more consolidated structure for further characterization. Figure 1 depicts the process of making *A. terreus*/SA composite beads.

### 2.4. A. terreus/SA Composite Beads Characterization

#### 2.4.1. Water Uptake Capacity

The ability of adsorbent beads (i.e., *A. terreus*/SA composite beads) to uptake water was identified to reveal the absorption behavior and interaction of beads with water. This test was conducted using filter paper to eliminate any surface moisture from the beads and then record the wet weight of the beads (*W_w_*, g). Then, the dry weight of these beads was recorded after drying in an oven at 50 °C for 2 days (*W_d_*, g). Finally, the difference in weight before and after drying (*W_a_*, g) was obtained from the following Equation (1):

(1) $$W_{a}=W_{w}-W_{d}/W_{d}$$

where *W_w_* (g) means the composite beads’ wet weight and *W_d_* (g) indicates the composite beads’ dry weight.

#### 2.4.2. Adsorption Experiments

Many factors influence ARS adsorption, including the mass ratios of *A. terreus* and SA, as well as pH, temperature, contact time, and initial ARS concentration. To study the *A. terreus*/SA composite adsorption effect on ARS, 5 ratios of *A. terreus*/SA composite beads as 0%, 10%, 20%, 30%, and 40% were used as adsorbents. Then, 15 beads of each composite, weighing 25 mg were placed in 50 mL of ARS solution containing 100 mg/L at pH 7. The beads were prepared with almost the same weight to maintain a uniform mass. Then, the ARS solution with beads was put in a shaker incubator at 25 °C with a speed of 150 rpm overnight. After that, the filtrate was attained and analyzed by atomic absorption spectrophotometry to determine the concentration of the remained dye after adsorption. Then, the adsorbent (i.e., composite beads) was collected after filtration and washed with distilled water. Afterward, these beads were dried overnight in a hot air oven at 40 °C and weighed again. The adsorption capacity (qe, mg/g) and removal (%) were determined by Equations (2) and (3), respectively:

(2) $$\mathrm{qe}\;(\mathrm{mg}/g)\;=\frac{\left(C_{0\;}-C_{F}\right)V}{M}$$

(3) $$Removal\;\left(\%\right)=\frac{C_{0\;}-C_{F}}{C_{0\;}}\times 100$$

where *C*_0_ signifies the initial concentration (mg/L), *C_F_* denotes the final concentration (mg/L), *V* (L) represents the aqueous medium volume, and *M* is the dry weight of composite beads (g).

For the following experiments, including pH, temperature, contact time, and initial ARS concentration, the composite bead ratio with the highest ARS adsorption behavior was nominated to conduct these experiments. The impact of the pH of the ARS solution was considered within 1–10. At the same time, the point of zero charge (PZC) was determined. PZC is defined as the pH value of the solution at which the biosorbent surface charge is equivalent to zero [36]. The PZC value of the ideal nominated ratio of *A. terreus*/SA composite beads was determined at the initial pH values (pH_i_) of 3–12. After that, the final pH (pH_f_) of 100 mg of composite beads mixed with 10 mL of potassium nitrate (0.1 mol/L) was measured after incubating for 24 h at 25 °C. Finally, the pH_i_ values were plotted versus pH_f_-pH_i_ to obtain the PZC value from the intersection of the drawn line with the X-axis on the graph. Also, the effect of temperature on the adsorption efficiency was tested at 15, 25, 35, and 45 °C. The contact time effect on adsorption capacity was determined every 30, 60, 90, 120, 150, and 180 min.

Moreover, different concentrations of ARS, ranging from 40, 60, 80, 100, 120, 140, and 160 mg/L, were investigated. This experiment with ARS concentrations was used to determine the adsorption isotherm. A kinetic analysis was carried out by measuring the concentration of ARS in the samples. A T70 spectrophotometer was employed to measure the ARS concentrations in the supernatant at 432 nm wavelength. 432 nm was nominated as the most appropriate and fixed lambda value after performing a spectral scan over a range of pH values without observing significant deviations.

#### 2.4.3. Reusability Experiments

Like the previous adsorption experiments, the reusability experiments were conducted on the optimum composite beads with the highest adsorption efficiency. The reusability experiments were conducted in four stages. Firstly, the used *A. terreus*/SA composite beads were rinsed in distilled H_2_O many times to get rid of any contaminants on the surface. Secondly, they were submerged in acidic ethanol (0.1 M HCl + 80% ethanol) to facilitate desorption. Thirdly, the composite beads were shaken at 35 °C using a shaker incubator for 6 h to enhance the desorption process (i.e., cleaning the composite beads). Finally, the composite beads were thoroughly washed with distilled H_2_O to neutralize any remaining acid.

### 2.5. Statistical Analysis

The one-way analysis of variance (ANOVA) at the *p* < 0.05 level was applied to compare the difference, and the Tukey test was performed (version 8.0 of GraphPad Prism). All results were performed in triplicate.

### 2.6. Composite Beads Characterization

The ideal composite bead ratio was characterized according to the optimum adsorption efficiency. More specifically, the characterization of morphological features and functional groups was performed on SA and the ideal nominated sample of *A. terreus*/SA composite beads (before and after the ARS adsorption process). The morphological features of prepared gel beads were detected using scanning electron microscopy (SEM; JSM-6510 LA, JEOL, Tokyo, Japan) after drying and covering with a nanometer-sized gold layer. Moreover, Fourier-transform infrared spectroscopy (FTIR; VERTEX 70, Bruker, Mannheim, Germany) was employed in the wavenumber range from 400 to 4000 cm^−1^ to investigate the functional groups of the composite beads.

### 2.7. Comparison with Previously Studied Adsorbents

The adsorption capacity of the recently prepared composite beads was compared to the different types of previously studied adsorbents for the same adsorbate (i.e., ARS dye). This comparison was investigated to evaluate the applicability of the addressed composite beads as a reliable adsorbent.

## 3. Results and Discussion

### 3.1. Fungal Identification

**Figure 2:**
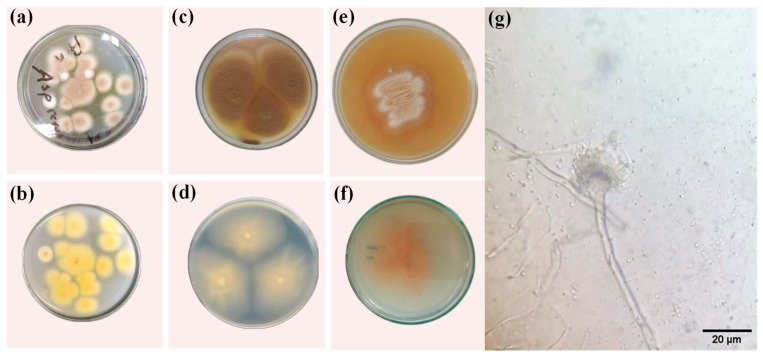
Colony morphology of *A. terreus* on PDA plates ((**a**) front, (**b**) backside), MEA plates ((**c**) front, (**d**) backside), SDA plates ((**e**) front, (**f**) backside), and (**g**) optical microscopic images showing hypha and the chain of conidia.

The isolated fungus grew for 5 days on the PDA petri dish at 28 °C and produced light-brown colonies at the front (Figure 2a) with bright-yellow color at the back (Figure 2b). However, the fungal colonies cultured on the SDA media plate under the same conditions had a buff color at the front (Figure 2c) and brown-yellow color at the back (Figure 2d). Furthermore, the fungus colonies on the MEA medium were dark orange (Figure 2e) with a light-yellow color on the back (Figure 2f). Microscopically, a compact columnar conidial head, transparent, soft long conidiophore, spherical to semi-spherical vesicles, and spherical conidia were observed, illustrating all features of the isolated fungus related to *A. terreus* [37]. Moreover, the fungus identification was validated by presenting a BLAST query of internal transcribed spacer (ITS) sequences on NCBI (https://blast.ncbi.nlm.nih.gov/Blast.cgi, accessed on 25 February 2023), and this identification confirmed that the fungus is *A. terreus* (MH865977.1).

### 3.2. Composite Beads Characterization

#### 3.2.1. Water Uptake Capacity

**Figure 3:**
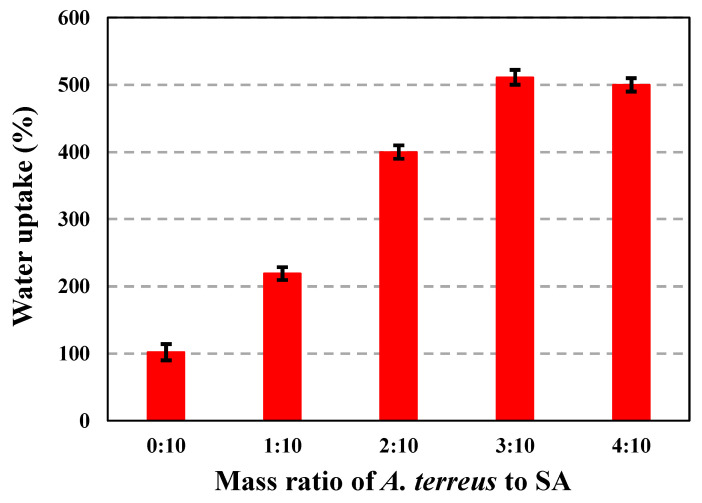
The ability of different mass ratios of *A. terreus*/SA to water uptake.

Following the weight loss before and after drying up, water carried most of the weight in the composite beads. Figure 3 depicts the capacity of various beads to absorb moisture. The proportions of water uptake for *A. terreus*/SA-0%, *A. terreus*/SA-10%, *A. terreus*/SA-20%, *A. terreus*/SA-30%, and *A. terreus*/SA-40% were 102, 219, 400, 511, and 500%, respectively. With the increase in the mass of *A. terreus*, the water uptake of composite beads increases by up to 30%. This can be attributed to the high water retention of *A. terreus* biomass. However, at 30%, *A. terreus* biomass becomes saturated and its water retention reaches a stable status. This makes *A. terreus* biomass act like a barrier, preventing further water uptake. Hence, the water content of the *A. terreus*/SA composite beads decreases slightly from 511 to 500% as the *A. terreus* powder content increases from 30 to 40%. Compared to the previous study [38], the water uptake capacity of the composite beads in our study is lower, possibly due to manufacturing issues affecting the subsequent adsorption results.

#### 3.2.2. Adsorption Studies

##### Influence of Mass Ratios on ARS Adsorption

**Figure 4:**
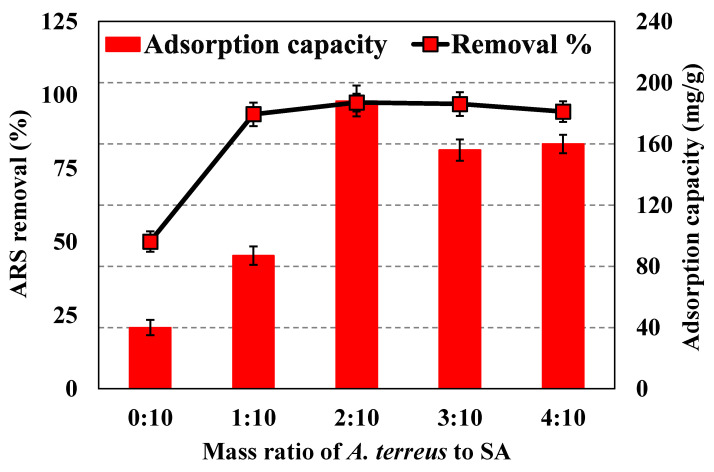
ARS adsorption behavior of 25 mg of different composite beads (0:10, 1:10, 2:10, 3:10, and 4:10) at a concentration of 100 mg/L and pH 7 at room temperature.

The effect of different mass ratios of *A. terreus* to SA on the ARS adsorption was studied to nominate the optimum ratio sample of composite beads necessary to complete the other adsorption experiments and characterization techniques. Figure 4 shows that the *A. terreus* powder-free SA has a low removal% of ARS. Also, it was found that the adsorption capacity of *A. terreus*/SA composite beads increases as the content of *A. terreus* powder increases. This indicates that adding *A. terreus* powder to SA significantly influences ARS adsorption. The results indicate that *A. terreus*/SA-20% has the highest ARS removal efficiency and adsorption capacity of 97.40% and 188 mg/g, respectively. The pure SA beads have the lowest ARS removal efficiency and adsorption capacity of 97.40% and 40 mg/g, respectively. Therefore, the *A. terreus*/SA-20% composite beads have the optimum mass ratio of *A. terreus* to SA (i.e., 20%) for the subsequent adsorption analysis and characterization techniques.

##### Influence of pH on ARS Adsorption

**Figure 5:**
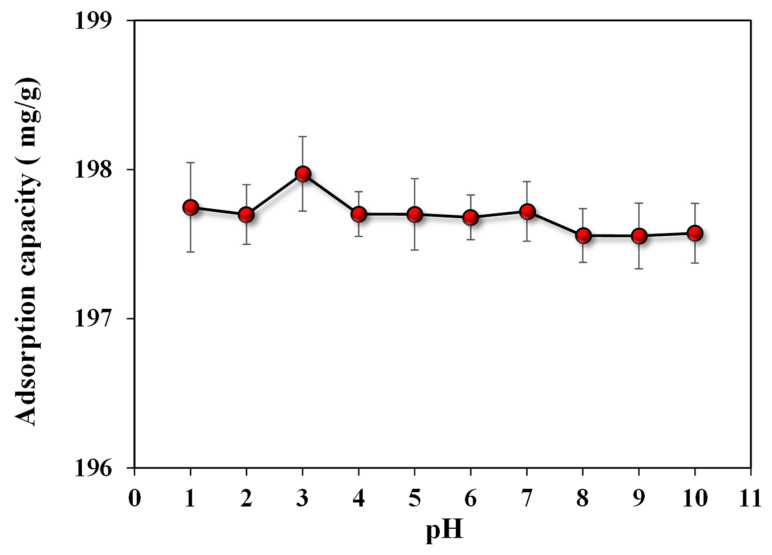
Influence of pH (1–10) on adsorption capacity (mg/g) of 25 mg *A. terreus*/SA-20% at a concentration of 100 mg/L at room temperature.

The surface charge of adsorbent and adsorbate can be affected by the pH of the solution via protonation and deprotonation mechanisms [39]. The surface charge influences adsorption by causing attraction or repulsion between the adsorbent material and the adsorbate. In our case, a low pH value is favorable for dye removal because it raises the positively charged sites, favoring the adsorption of anionic dyes by an electrostatic attraction [40]. Hydroxyl groups (−OH) in SA are generally protonated at lower pH, which facilitates the adsorption of anionic dyes [41]. Moreover, ARS is an anionic dye with a negative surface charge. Therefore, the Coulomb force between anionic ARS and cationic adsorbents may explain why it is more easily adsorbed in acidic environments [42]. In a broad pH range, the *A. terreus*/SA-20% composite beads exhibited optimal adsorption effects on ARS, as shown in Figure 5. The high specific surface area and abundance of functional groups in *A. terreus*/SA-20% composite beads provide high adsorption potential. The adsorption capacity of *A. terreus*/SA-20% composite beads exceeded 197 mg/g in the pH range of 1–10.

**Figure 6:**
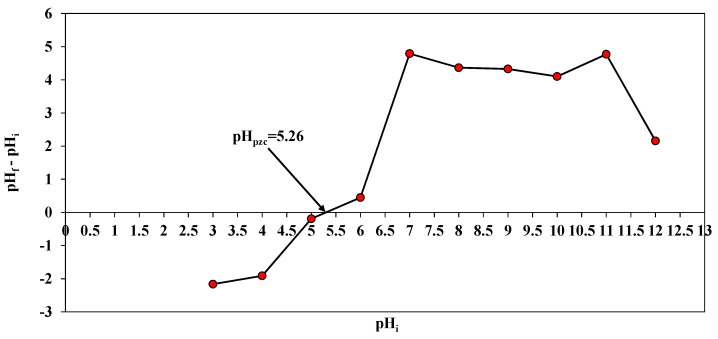
pH_pzc_ of *A. terreus*/SA-20% composite beads.

The adsorption of ARS was attributed to the electrostatic attraction between the positive functional groups of *A. terreus*/SA composite beads, such as the amine and hydroxyl groups of ARS dye. The results show a maximum adsorption capacity of 197.97 mg/g at pH 3. It can be because the adsorbate surface carries a positive charge at an acidic pH [43]. Possibly, this is the result of increased protonation of the weak base group on *A. terreus*/SA composite beads at lower pH, which increased dye adsorption. The pH_pzc_ of *A. terreus*/SA composite beads was 5.26 based on the pH_i_ vs. pH_f_–pH_i_ plot (Figure 6). Anionic dyes are highly favored at pH values below pH_pzc_ for their adsorption [44]. The total positive charges on the surface of *A. terreus*/SA composite beads equal the total negative charges at this pH value. In other words, the surface of the *A. terreus*/SA composite beads is positively charged when the pH is less than pH_pzc_ and negatively charged when the pH is more than pH_pzc_.

##### Influence of Temperature on ARS Adsorption

**Figure 7:**
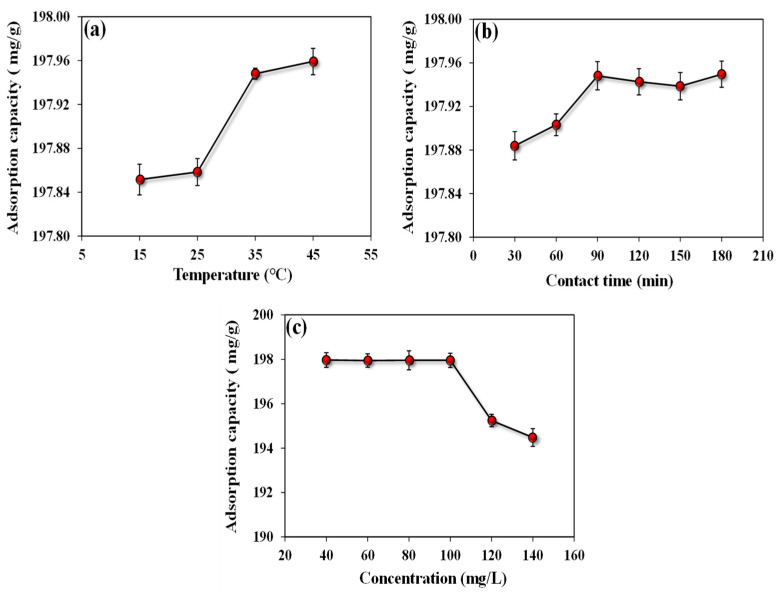
Relation between adsorption capacity (mg/g) of 25 mg *A. terreus*/SA-20% and each of (**a**) temperature (°C), (**b**) contact time (min), and (**c**) initial concentration (mg/L) at a concentration of 100 mg/L and pH 3 at room temperature.

The adsorption process is greatly influenced by temperature. Heat exchange occurs in conjunction with adsorption, and most adsorption processes are endothermal. A relatively wide range of temperature variations in the dye wastewater emitted from the commercial production process impacts the adsorption of the *A. terreus*/SA-20% composite beads. Figure 7a shows that *A. terreus*/SA-20% composite beads have a high adsorption capacity ranging from 197.85 to 197.95 mg/g at temperatures ranging from 15 °C to 45 °C, respectively. Therefore, 45 °C represents the optimal temperature at which the ARS adsorption capacity reaches its maximum value (i.e., 197.95 mg/g). As for most adsorbents, the adsorption capacity of composite beads increases with a temperature rise. This indicates that higher temperatures stimulate the contact of dye particles with *A. terreus*/SA-20% composite beads, causing the dye particles to move more actively. As a result, *A. terreus*/SA-20% composite beads are effective for ARS adsorption over a wide temperature range.

##### Influence of Contact Time on ARS Adsorption

Figure 7b depicts the difference in the adsorption capacity rate with time. The results illustrate that the adsorption capacity gradually increases with time. More explicitly, the adsorption capacity reaches 197.94 mg/g at 90 min, then decreases very slowly, reaching 197.93 mg/g at 150 min, and finally, it reaches the maximum value (197.95 mg/g) at 180 min. At the beginning of adsorption, the high concentration of ARS in the aqueous solution works as the driving energy for ARS adsorption, which is supported by a concentration gradient. The adsorbent also has a lot of active sites on its surface, which facilitates the adsorption procedure. As the active sites on the surfaces of adsorbents become saturated later in the process, adsorption and intra-particle diffusion are delayed, and the adsorption rate gradually decreases. The following section explored adsorption kinetics further to explain the connection between the adsorption process and time.

##### Influence of Initial ARS Concentration on Adsorption

Generally, the effect of the initial ARS concentration on adsorption capacity is influenced by the concentration gradient and attraction force of the adsorbate [45]. Figure 7c illustrates the influence of the initial ARS concentration (i.e., 40, 60, 80, 100, 120, and 140 mg/L) on adsorption. As the initial ARS concentration was less than 100 mg/L, the ARS adsorption capacity was high but almost stable, ranging between 194 and 197 mg/g. For less than 100 mg/L, the available adsorption sites are plentiful, and adsorption can reach equilibrium quickly; thus, the adsorption efficiency is high, with little relationship to the initial concentration [46]. Otherwise, the adsorption capacity (mg/g) dramatically decreases with the increase in the initial concentration to more than 100 mg/L. This can be attributed to the critical deficiency in the active sites of adsorption on the composite due to blocking these sites. The effect of initial concentration on adsorption outcomes can be investigated further in the following isotherm section.

### 3.3. Adsorption Kinetics

**Figure 8:**
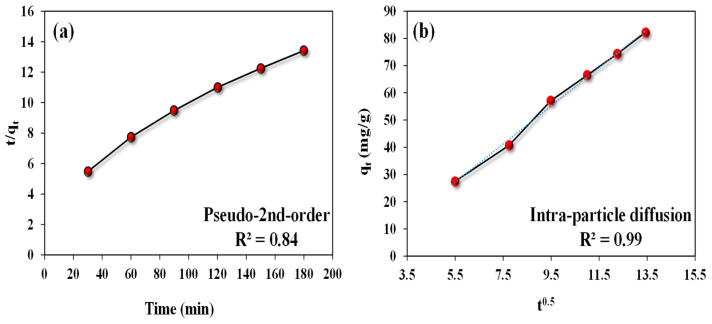
Kinetic studies of ARS uptake by 25 mg *A. terreus*/SA-20% composite beads at a concentration of 100 mg/L and pH 3 at room temperature, (**a**) Pseudo-second-order model for ARS uptake and (**b**) Intra-particle diffusion model for ARS uptake.

The kinetics of ARS adsorption by *A. terreus*/SA composite beads were studied using two standard models: the pseudo-second-order and inter-particle diffusion models. These linear forms of models and determined parameters are listed in Table 1. As illustrated in Table 2, the experimental results of ARS adsorption are more compatible with the intra-particle diffusion model than the pseudo-second-order model. This can be validated by the R^2^ value of the intra-particle diffusion model (0.99), which is greater than that of the pseudo-second-order model (0.84) by 16.67%. This indicates that the possibility of inner pore diffusion is the most prominent [47]. The pseudo-second-order model accurately describes the ARS adsorption process by *A. terreus*/SA composite beads (adsorbent) (Figure 8a), as evidenced by R^2^ (0.84) and q_e_ (96.15 mg/g) for the adsorbent (Table 2). Otherwise, the plotting of qt (mg/g) versus t^0.5^ (min^0.5^) in the intra-particle diffusion model is presented in Figure 8b. This relation does not pass through the origin point (0, 0), implying that the adsorption process is dominated by intra-particle diffusion (i.e., internal diffusion) [48]. Therefore, the C constant equals 11.53 (Table 1), indicating that the boundary layer affects adsorption.

### 3.4. Adsorption Isotherms

**Figure 9:**
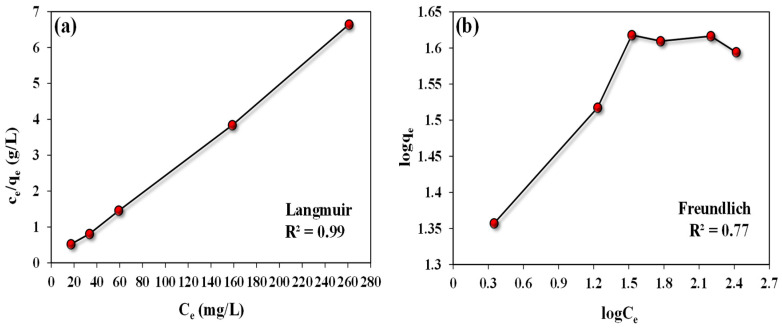
Isotherm studies for ARS uptake by *A. terreus*/SA-20% composite beads: (**a**) Langmuir model and (**b**) Freundlich model.

Langmuir and Freundlich isotherm models were employed to examine the interactive nature between adsorbate (ARS) and adsorbents (*A. terreus*/SA composite beads) and collect data about the surface characteristics of the adsorbent and their attraction properties to the studied adsorbate. Table 3 shows the linear expressions of these models as well as their estimated parameters. Generally, the Langmuir isotherm assumes that the adsorption process is uniform and happens at certain active spots on the adsorbent surface with identical energy. Through this adsorption method, the adsorbate forms a monolayer on the adsorbent surface [51]. On the other hand, the Freundlich isotherm supposes that the adsorbate will be adsorbed in several layers on the accessible active sites on the adsorbent surface (i.e., a heterogeneous adsorption process) [52]. Linear plots were figured between C_e_/q_e_ (g/L) vs. Ce (mg/L) and log q_e_ vs. log C_e_ to deduce the Langmuir and Freundlich parameters, respectively (Figure 9a,b). The findings of the determined coefficients for the two models reveal that the Langmuir model represents the adsorption data of ARS by the *A. terreus*/SA composite beads better than the Freundlich isotherm, as illustrated in Table 3. This is verified by the R^2^ value of the Langmuir model (0.99), which is higher than that of the Freundlich model (0.77) by 28.57%. More specifically, the maximum adsorption capacity of the addressed ARS (q_m_) in the Langmuir model is 192.30 mg/g compared to its counterpart (K_F_), which is equal to 22.91 mg/g in the Freundlich model. As a result, the ARS dye is evenly adsorbed on the homogeneous adsorption surface of the *A. terreus*/SA composite beads. Additionally, the ARS dye ions are subsequently adsorbed to the positively charged and finite active sites of the *A. terreus*/SA composite beads surfaces, which are organized into a monolayer. At the same time, the K_L_ value (0.207 L/mg), which expresses the privileged adsorption of the ARS by the examined *A. terreus*/SA composite beads, supports the validity of the Langmuir equation in describing the ARS adsorption data (Table 4) [53].

### 3.5. Adsorption Mechanism

**Figure 10:**
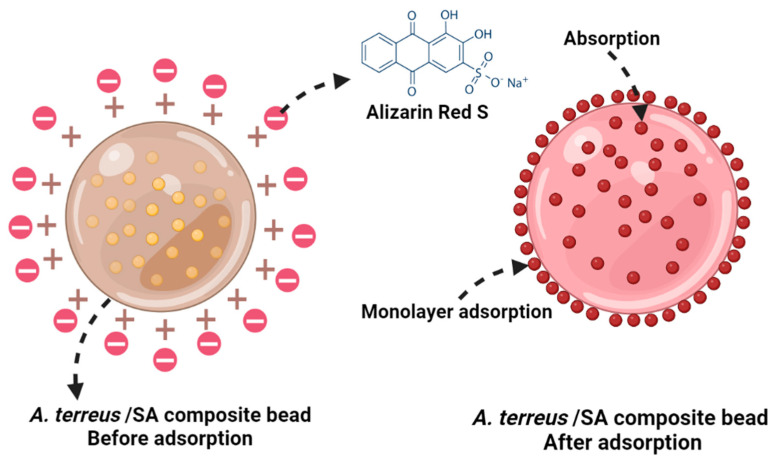
A simple schematic of the adsorption mechanism of Alizarin Red S by *A. terreus*/SA-20% composite beads.

Figure 10 illustrates the ARS uptake mechanism of the *A. terreus*/SA composite bead. ARS is an anionic dye with a sulfonate group (R–S(=O)_2_–O^−^) that carries a negative charge in the aqueous solution [56]. Complying with FTIR results, the uptake mechanisms can occur by electrostatic interactions and interior adsorption between the adsorbate molecules (i.e., ARS) and the functional groups, particularly carboxyl, hydroxyl, and amine, on the surface of the biosorbent. Additionally, the adsorbate particles are gradually diffused into the interior particles of the adsorbent (i.e., internal diffusion). This mechanism happens when there is saturation in the enriched surface monolayer in the adsorbent [57].

Moreover, the Langmuir model reveals that these functional groups on the adsorbent can work as active sites for the adsorbate. Therefore, it is predicted that the ARS adsorbed on the surface of *A. terreus*/SA composite beads cannot be damaged, and the structure of the ARS molecule will not change [58]. Subsequently, a suitable desorption method can enable it to be recovered and reused. It will be further investigated in the following reusability section.

### 3.6. Reusability

**Figure 11:**
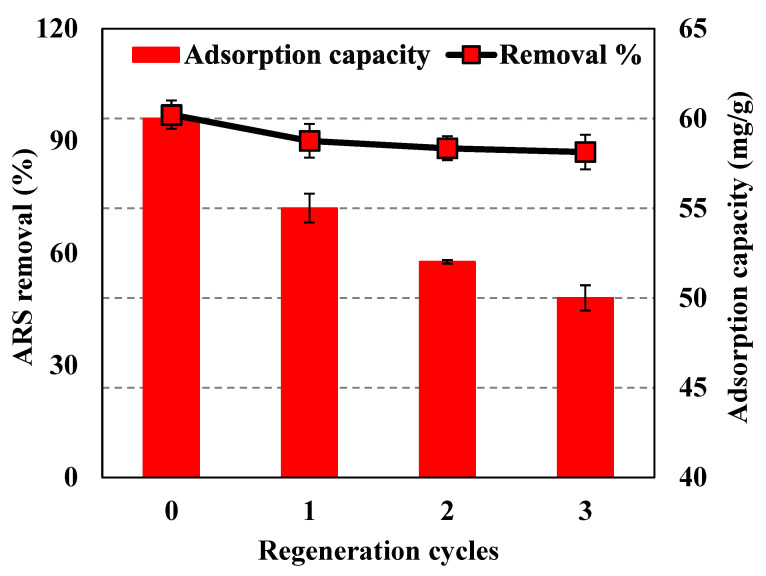
Reusability of *A. terreus*/SA-20% composite beads for ARS uptake.

The reusability of *A. terreus*/SA during the ARS adsorption process is essential for sustainable application on a realistic and commercial scale. Simple separation technology allows quick extraction of *A. terreus*/SA composite beads from the solution, enabling subsequent reuse and desorption. The reusability is characterized by the sorption-desorption cycles of the adsorbent (Figure 11). After the first cycle of adsorption and desorption experiments, *A. terreus*/SA-20% composite beads had a high effect on ARS adsorption, with removal% and adsorption capacity of 90% and 55 mg/g, respectively. After three cycles, the removal% and adsorption capacity of the *A. terreus*/SA composite beads declined to 87% and 50 mg/g, respectively. Therefore, the reusability process transforms *A. terreus*/SA composite beads into sustainable, eco-friendly adsorbent material. Finally, it was found that electrostatic interactions may be the predominant mechanism for decolorization at lower pH.

### 3.7. SEM and FTIR Characterization

**Figure 12:**
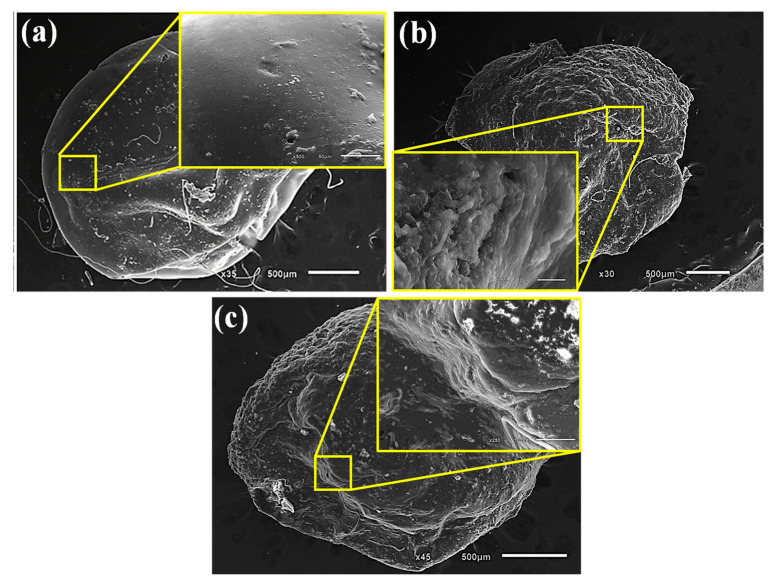
SEM images of (**a**) SA beads, (**b**) *A. terreus*/SA-20% composite beads before the ARS uptake, and (**c**) *A. terreus*/SA composite beads after the ARS uptake.

Figure 12 depicts the morphological description of air-dried SA beads and *A. terreus*/SA-20% composite beads before and after the adsorption of ARS. It was found that the surface morphology of SA beads is smooth with no observed undulations (Figure 12a). Before the ARS uptake, the surface of the *A. terreus*/SA-20% composite beads is rough and has protrusions, indicating that the *A. terreus* powder has a significant morphological effect on the SA surface (Figure 12b). In contrast, the *A. terreus*/SA-20% composite beads have wrinkles and an accordion-like multilayer structure after the ARS uptake (Figure 12c). More explicitly, the *A. terreus*/SA-20% composite beads can be formed due to the coating of *A. terreus* powder with SA film or adhering to its surface. This phenomenon is corroborated by previous work [59]. These SEM images indicate that the *A. terreus*/SA-20% has strong uptake properties.

**Figure 13:**
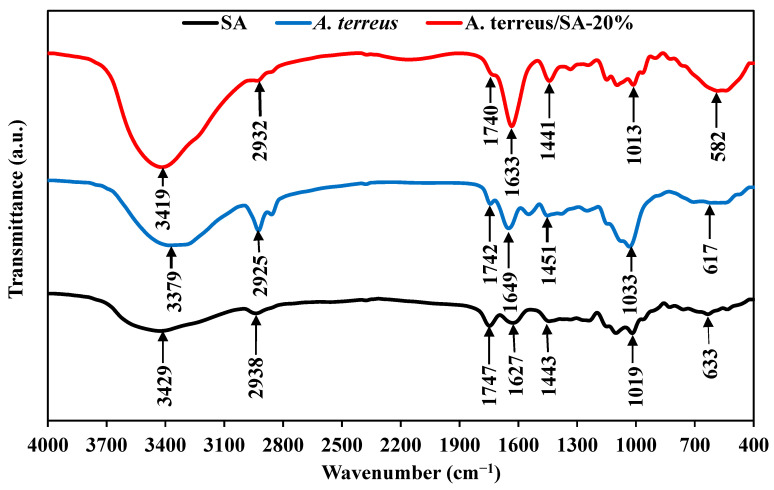
FTIR spectrum of SA beads, *A. terreus*, and *A. terreus*/SA-20% composite beads.

Figure 13 depicts the FTIR spectra of SA, *A. terreus* powder, and *A. terreus*/SA-20% composite beads. In FTIR analyses, the discussion was focused on the most effective bands, which work as active sites susceptible to the adsorption process. This can be clearly illustrated by observing the enlargement in the intensity of the effective bands of these functional groups, which are mainly responsible for adsorption in the newly formed composite beads. On the other hand, the less effective bands are represented by less intense and narrow peaks, which have minor impacts on adsorption. This can appear in the characteristic bands at 3429, 3379, and 3419 cm^−1^ related to the stretching of the hydroxyl groups (−OH) in SA, *A. terreus* powder, and *A. terreus*/SA-20% composite beads, respectively [60,61]. More specifically, the intensified and broader hydroxyl band at 3419 cm^−1^ in *A. terreus*/SA-20% composite beads is produced from the combination of its counterpart bands at 3429 and 3379 in SA and *A. terreus* powder, respectively. The same scenario occurred at the intensified and shifted bands of 1627 and 1441 cm^−1^, which indicates the symmetric C–O bond in carboxylate [62,63,64] in *A. terreus*/SA-20% composite beads compared to their corresponding bands at 1649 and 1633 cm^−1^, as well as 1443 and 1451 cm^−1^ in SA and *A. terreus* powder, respectively.

Moreover, there is an enlarged band with a slight change at 582 cm^−1^ in the composite beads, indicating the sulfonic acid group, due to the combination of the bands at 633 and 617 cm^−1^ in SA and *A. terreus* powder, respectively. All previous bands with higher intensity in the composite beads can be attributed to the integration between the bands found in SA and *A. terreus* powder. On the other hand, the peak at 2932 cm^−1^, which signifies the asymmetric stretching vibrations of the CH_2_ group in the composite beads, shows less intensity with a minimal move compared to its counterparts at 2938 and 2925 cm^−1^ [65]. Also, the peak at 1740 in the composite beads, which indicates C=O stretching [66], suffers from a reduction in intensity compared to those peaks at 1747 and 1742 cm^−1^ in SA and *A. terreus* powder, respectively. Regarding the small band at 1013 cm^−1^, which represents C−O stretching vibration [67] in the composite beads, it is shifted with lower intensity compared to the bands at 1019 and 1033 cm^−1^ in SA and *A. terreus* powder, respectively. All previous peaks with lower intensities in the composite beads indicate that SA has encapsulated *A. terreus* powder, decreasing the effect of some functional groups.

### 3.8. Comparison with Previous Studies

Table 5 compares the maximum adsorption capacity of *A. terreus*/SA composite beads with other adsorbents from previous studies after various treatment processes relative to the Langmuir isotherms. The *A. terreus*/SA composite beads show significant ARS adsorption capacity compared to other adsorbents, as shown in Table 5. Therefore, it can be applied as a potential alternative to common adsorbents for treating water polluted with ARS.

## 4. Conclusions

The findings of this study can represent the following statements:

In this work, the *A. terreus*/SA composite beads were successfully prepared with different ratios of *A. terreus* (i.e., 0%, 10%, 20%, 30%, and 40%) to sodium alginate (SA). As explained by the experimental results, the composite beads of *A. terreus*/SA-20% composite beads had the highest ARS adsorption capacity of 188 mg/g, which was compatible with the Langmuir isotherm model with q_m_ = 192.30 mg/g. According to the adsorption kinetics, the adsorption process matched well with pseudo-second-order and intra-particle diffusion models. This was congruent with the morphological and chemical features indicated by SEM and FTIR, respectively. Moreover, the *A. terreus*/SA-20% composite beads showed significant recycling for ARS in three cycles with a total removal rate of 87% and an adsorption capacity of 50 mg/g, lowering the adsorption cost. Finally, *A. terreus*/SA-20% beads are an innovative composite that can be employed as an eco-friendly, easily manufactured, inexpensive, and sustainable adsorbent compared to other conventional adsorbents.

## Figures and Tables

**Table 1:** Adsorption kinetics models for ARS uptake by *A. terreus*/SA-20% composite beads.

Kinetics Model	Linear	Parameters	Refs.
Pseudo-second-order	$\;\frac{t}{q_{t}}=\frac{1}{k_{2}q_{e}{}^{2}}+\frac{1}{q_{e}}t$	*K*_2_: Rate constant of the second-order adsorption (g/mg min).	[49]
Intra-particle diffusion	$q_{t}=k_{p}t^{0.5}+c$	*K_p_* (mg/g min^0.5^): Rate constant of intra-particle diffusion. *C* (mg/g): Intercept of the line.	[50]

**Table 2:** Parameters of kinetics models for ARS uptake by *A. terreus*/SA composite beads.

Kinetic Model	Parameters		
Pseudo-second-order	***q*_*e*_ (mg/g)**	***K*_2_ (g/mg min)**	**R^2^**
	96.15	0.00021	0.84
Intra-particle diffusion	***C* (mg/g)**	***K*_*p*_ (mg/g min^0.5^)**	**R^2^**
	11.53	5.94	0.98

**Table 3:** Isotherms models for ARS uptake by *A. terreus*/SA composite-20% beads.

Isotherms Models	Linear	Parameters	Refs.
Langmuir	$\frac{c_{e}}{q_{e}}=\frac{c_{e}\;}{q_{m}}+\frac{1}{k_{L}q_{m}}$	*C_e_* (mg/L): Remaining ARS concentration in solution. *q*_*e*_ (mg/g): Removed ARS at equilibrium. *q*_*m*_ (mg/g): Maximum adsorption capacity *k_L_* (L/mg): Langmuir constant of the isotherm.	[54]
Freundlich	$logq_{e}=logk_{f}+\frac{1}{n}logC_{e}$	*K_f_* (mg/g): Adsorption capacity of ARS. *n*: Heterogeneity factor.	[55]

**Table 4:** Parameters of isotherms models for ARS uptake by *A. terreus*/SA-20% composite beads.

Parameters	Isotherm Models	
	Langmuir	Freundlich
*q*_*m*_ (mg/g)	192.30	—
*K*_*L*_ (L/mg)	0.207	—
*K*_*F*_ (mg/g)	—	22.91
1/*n*	—	0.121
R^2^	0.99	0.77

**Table 5:** Comparison of the maximum adsorption capacity (q_m_, mg/g) of *A. terreus*/SA composite beads to remove ARS with other previously studied adsorbents.

Adsorbent	q_m_ (mg/g)	Ref.
Biomass-based activated carbon	91.69	[32]
Multi-walled carbon nanotube	45.39	[34]
Carbonaceous adsorbents derived from textile cotton waste	74.00	[68]
Nickel and iron-based metal-organic frameworks	176.68	[69]
Polyalthia longifolia-based alumina composites	25.06	[70]
Activated carbon/γ-Fe2O3 nano-composite	108.69	[71]
Activated clay modified by iron oxide	32.70	[33]
Graphene oxide–Attapulgite composite	55.81	[72]
*A. terreus*/SA composite beads	192.30	Current study

## Data Availability

All data available through the manuscript.
